# Shifting the microbiome of a coral holobiont and improving host physiology by inoculation with a potentially beneficial bacterial consortium

**DOI:** 10.1186/s12866-021-02167-5

**Published:** 2021-04-28

**Authors:** Ying Zhang, Qingsong Yang, Juan Ling, Lijuan Long, Hui Huang, Jianping Yin, Meilin Wu, Xiaoyu Tang, Xiancheng Lin, Yanying Zhang, Junde Dong

**Affiliations:** 1 CAS Key Laboratory of Tropical Marine Bio-resources and Ecology, Guangdong Provincial Key Laboratory of Applied Marine Biology, South China Sea Institute of Oceanology, Chinese Academy of Sciences, Guangzhou, 510301 China; 2 Southern Marine Science and Engineering Guangdong Laboratory (Guangzhou), Guangzhou, 511458 China; 3 Ocean school, Yantai University, Yantai, 264005 China; 4 Tropical Marine Biological Research Station in Hainan, Chinese Academy of Sciences and Hainan Key Laboratory of Tropical Marine Biotechnology, Sanya, 572000 China; 5 Innovation Academy of South China Sea Ecology and Environmental Engineering, Chinese Academy of Sciences, Guangzhou, 510301 China; 6 University of Chinese Academy of Sciences, Beijing, 100049 China

**Keywords:** *Pocillopora damicornis*, Beneficial bacterial consortium, High-throughput sequencing, *Endozoicomonas*

## Abstract

**Background:**

The coral microbiome plays a key role in host health by being involved in energy metabolism, nutrient cycling, and immune system formation. Inoculating coral with beneficial bacterial consortia may enhance the ability of this host to cope with complex and changing marine environments. In this study, the coral *Pocillopora damicornis* was inoculated with a beneficial microorganisms for corals (BMC) consortium to investigate how the coral host and its associated microbial community would respond.

**Results:**

High-throughput 16S rRNA gene sequencing revealed no significant differences in bacterial community α-diversity. However, the bacterial community structure differed significantly between the BMC and placebo groups at the end of the experiment. Addition of the BMC consortium significantly increased the relative abundance of potentially beneficial bacteria, including the genera *Mameliella* and *Endozoicomonas*. Energy reserves and calcification rates of the coral host were also improved by the addition of the BMC consortium. Co-occurrence network analysis indicated that inoculation of coral with the exogenous BMC consortium improved the physiological status of the host by shifting the coral-associated microbial community structure.

**Conclusions:**

Manipulating the coral-associated microbial community may enhance the physiology of coral in normal aquarium conditions (no stress applied), which may hypothetically contribute to resilience and resistance in this host.

**Supplementary Information:**

The online version contains supplementary material available at 10.1186/s12866-021-02167-5.

## Background

Stony corals are marine invertebrates in the class Anthozoa, phylum Cnidaria, and are complex holobionts composed of the coral polyps and a rich microbiome including dinoflagellates, bacteria, archaea, fungi, and viruses [1, 2]. Coral holobionts are excellent ecosystem engineers that build spectacular coral reefs by continuously secreting and depositing calcium carbonate [3]. Coral reefs are one of the most complex and diverse ecosystems worldwide and are mainly distributed in shallow tropical and subtropical oceans. Coral reefs supply food and habitats to sustain diverse and abundant marine life [4]. In addition, this valuable ecosystem supports millions of people, both socially and economically, via coastal protection, provision of marine food, recreation and tourism, and pharmaceuticals [4, 5]. However, under the influence of ocean warming, ocean acidification, high ultraviolet radiation, and local human interference (overfishing and pollution), coral reefs globally are facing unprecedented degradation pressure [6–8]. One marked example is the bleaching of coral on the Great Barrier Reef. Five massive bleaching events occurring in 1998, 2002 2016, 2017 and 2020, has struck all three regions of the Great Barrier Reef. The coral bleaching event of 2016, which mainly affected the northern region of the Great Barrier Reef, was the most extensive and severe, and the proportion of reefs that experienced extreme bleaching was over four times that of the events in 1998 or 2002 [9, 10]. The bleaching in 2017 was most intense in the middle section of the Great Barrier Reef, while the 2020 bleaching was more widespread than earlier events and affected large parts of the southern sectors of the Great Barrier Reef [11, 12]. In addition, reefs previously considered as refugues have been threatened in recent years. For example, South Atantic reefs, which had previuously escaped multiple thermal stress, experienced their first mass die-off events in 2019 [13]. Without positive conservation measures, coral reef bleaching will cause further deterioration, bringing significant and unimaginable consequences for humans and other organisms [14, 15].

Coral restoration programs are currently classified into two categories: active and passive restoration. Active restoration is the active intervention of humans to recuperate or improve the state of the ecosystem, while passive restoration aims to reduce or eliminate anthropogenic effects on the ecosystem to achieve natural recovery [16]. Existing passive restoration measures predominantly include creating marine protected areas (MPA), adopting legislation and enforcement acts, and reducing exploitation of reef resources [17]. Although MPA numbers are increasing and other measures have been implemented, only limited success has been achieved, and coral reefs continue to disappear at alarming rates [9]. Therefore, the urgent need for coral reef restoration led to the concept of active restoration. This intervention process incorporates many research methods such as coral gardening and ecological engineering techniques, assisted approaches, coral epigenetics, and coral chimerism [18]. Coral gardening is one of the most common active restoration methods, and involves transplanting coral fragments (asexual) or cultivated coral seedlings (sexual) into degraded reefs or artificial reef structures [17]. However, application of coral asexual fragments places inherent practical constraints on the conserved genetic diversity and the achieved spatial scale, and sexual reproduction is limited by many factors, such as the time of coral broadcast-spawning or coral brooding, the coral seedling populations, and low rates of larvae attachment, metamorphosis, and survival [19]. Some advancements in ex situ spawning and controlling the spawning times along with increasing growth and post sttlement survival have been reported [20, 21], but other active restoration measures remain at a conceptual level or in initial laboratory trials. Thus, more research is required to clarify existing or potential problems to facilitate the active restoration of coral.

The development of genome sequencing and meta-omics technologies has further clarified relationships between microorganisms and their hosts. Accumulating evidence indicates that the microbiome plays key roles in host health, with involvement in energy metabolism, nutrient cycling, and immune system formation [22]. Hence, microbiomes have been engineered to improve hosts and their surrounding ecological environments. For example, fecal microbiota transplantation in humans was highly effective in treating gastrointestinal disease caused by recurrent *Clostridium difficile* infection [23], and showed great potential for treating irritable bowel syndrome [24]. In broilers, *Bacillus subtilis CH16* promoted daily weight gain and reduced food conversion rates [25]. Furthermore, the application of probiotics in plants alleviated drought stress in *Arabidopsis thaliana*, promoted growth and yield in *Arachis hypogaea*, and inhibited infection in sugar beet [26–28]. The results achieved in many complex organisms, coupled with the close relationships that exist between the coral microbiome, coral health, and stability of reef ecosystems, suggest that the principle of engineered microbiomes could be applied to recover lower organisms such as coral [29].

Peixoto et al. proposed the Beneficial Microorganisms for Corals (BMC) framework in 2017, based on the Coral Probiotic Hypothesis, and this hypothesis initially helped explain the evolutionary success of coral and is now also used in coral microbial research [30–33]. Combining these two concepts provided new approaches for developing and using BMC consortia (i.e., the symbiotic microorganisms involved in maintaining and protecting the physiological balance of coral) as environmental probiotics to manipulate the microbiome, reverse dysbiosis, and restore and protect coral reefs [30, 31]. Application of BMC consortia is in its infancy, but some encouraging results have been reported. For example, *Vibrio coralliilyticus* inoculated into coral destabilized the host microbiome, while simultaneous inoculation of *Halobacteriovorax* predator (a unique bacterial predator that can prey on *vibrio*) mitigated the negative effects on the coral [34]. Adding a bacterial consortium to *Pocillopora damicornis* lessened the coral bleaching caused by high temperature stress [35], while an oil bioremediation-potential BMC consortium preserved the photochemical ability of coral that can be decreased by oil pollution [36]. Additionally, Damjanovic et al. evidenced the feasibility of coral microbiome manipulation using coral early recruits [37]. These studies indicated the great potential of BMC for improving the health of coral under stress. However, how a BMC consortium affects the physiological state of its coral host and the structure of the coral microbiome in a healthy environment has yet to be eclucidated. Nitrogen and phosphorus are essential elements for coral reef productivity. However, nitrogen is regarded as a proximate limiting nutrient for primary production in coral reefs, and phosphorus is unavailable to the coral reef organisms due to this element existing in the form of highly insoluble macromolecular phosphorus [38, 39]. In this study, coral physiology and microbial community structure were analyzed after adding a potential beneficial bacterial consortium, comprising members that were selected based on their potential nitrogen-fixation and phosphate-solubilization abilities. The bacteria consortium contributed to the energy reserves and calcification ability of *Pocillopora damicornis*, possibly by shifting the coral microbial community structure.

## Results

### Effects on coral physiology

**Fig. 1:**
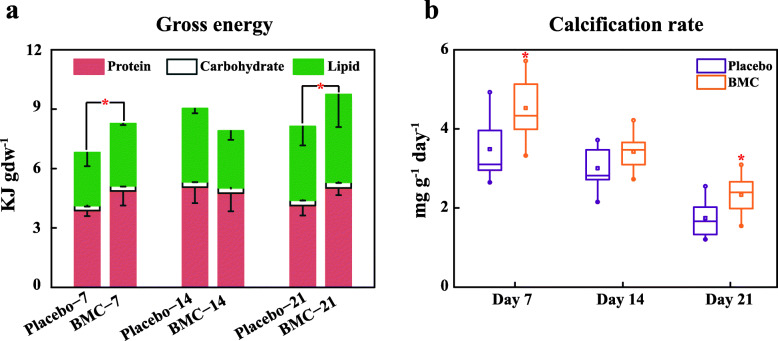
Coral gross energy reserves and calcification rates following exposure to a bacterial consortium. **a** Changes in coral gross energy reserves on days 7, 14 and 21. **b** Changes in average daily calcification rates in the placebo (purple boxes) and BMC (orange boxes) groups. * and ** indicate significant differences at *P* < 0.05 and *P* < 0.01, respectively. Bars represent the standard deviation of the mean. Number of coral fragments: gross energy reserves, *n* = 6; calcification rate, *n* = 9

Lipid and carbohydrate concentrations of the coral samples exposed to BMC did not significantly differ from those of the placebo groups throughout the experiment. However, protein concentrations of the BMC groups significantly increased by 26 and 21% compared with those of the placebo group on day 7 (*P* = 0.013) and day 21 (*P* = 0.025), respectively (Fig. S1). Moreover, the gross energy reserves (protein, lipids, and carbohydrates) in the BMC group were significantly higher (20%) than those of the placebo group on day 21 (*P* = 0.02; Fig. 1a). Calcification rates were also significantly higher in BMC-treated corals compared with the placebo groups on days 7 and 21 (Fig. 1b). The calcification rate of the BMC group was 30% higher than that of placebo group on day 7 (*P* = 0.001) and 33% higher than that of the placebo group on day 21 (*P* = 0.049). Throughout the time course of the experiment, chlorophyll-a concentrations did not significantly differ between the BMC and placebo groups, but the chlorophyll-a concentration of the BMC group gradually increased and exceeded that of the placebo group on day 21 (Fig. S2a). Photosystem II maximal efficiency (Fv/Fm) of the BMC group did not significantly differ from that of the placebo group throughout the experiment (Fig. S2,2,2b).

### 16S rRNA gene composition, diversity and community structure

**Fig. 2:**
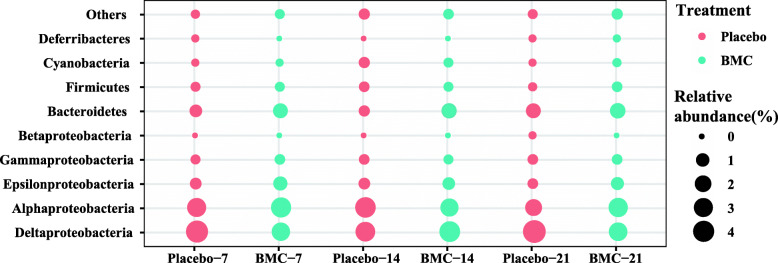
Relative abundances of the coral microbiome of placebo and BMC groups at the phylum level (except Proteobacteria, which is represented at the class level). Bubble size indicates relative abundance, and different colors represent different treatments. Numbers 7, 14 and 21 represent days 7, 14 and 21, respectively. Placebo: received no bacterial consortium; BMC: received the bacterial consortium

After processing, high-quality 16S rRNA gene sequences (420 bp–450 bp) yielded from the 36 fragments of *Pocillopora damicornis* were normalized to 15,000 sequences per sample. All sequences could be assigned to 9239 operational taxonomic units (OTUs) using VSEARCH [40] with grouping based on 97% similarity level. The 16S rRNA gene sequences retrieved from all coral samples were classified into 45 bacterial and two archaeal phyla. Sequences related to bacteria within the phylum Proteobacteria were most abundant, followed by the phylum Bacteroidetes, then Firmicutes and Cyanobacteria (Fig. 2). Within the phylum Proteobacteria, the two predominant classes were Deltaproteobacteria and Alphaproteobacteria, with an average of 16 and 12% of the sample sequences, respectively. The relative abundance of Alphaproteobacteria in BMC-treated corals did not significantly change over the three time points examined. However, the relative abundance of Deltaprobacteria was significantly lower in BMC-treated corals than in placebo-treated corals on day 21 (*P* = 0.0187; Additional file 3). Thus, adding the bacterial consortium affected the coral-associated microbial community composition.

The α-diversity was analyzed via the number of OTUs and Chao1, Shannon-Wiener’s and Simpson evenness indexes. Student’s t-test was used to assess statistical significance of the differences between the placebo and BMC group samples. BMC treatment did not significantly influence the bacterial richness and evenness (*P* > 0.05; Table 1). Three nonparametric multivariate statistical methods (Multi Response Permutation Procedure [MRPP], Adonis, and Analysis of similarities [ANOSIM]) were performed using the Bray-Curtis dissimilarity index to explore whether the bacterial consortium affected the taxonomic composition and bacterial community structure of the coral. The microbial community structure differed significantly between the placebo- and BMC-treated corals on day 21 (*P* < 0.05; Table 2), even though there was no clear separation between placebo and BMC groups on days 7, 14 and 21 in Principal Coordinates Analysis (PCoA) plots (Fig. S3).

**Table 1:** Richness and diversity indices of the 16S rRNA gene from coral (*n* = 6) in three times point (expressed as the mean ± standard deviation [SD])

	Day 7			Day 14			Day 21		
	Placebo	BMC	***P***	Placebo	BMC	***P***	Placebo	BMC	***P***
**Chao1**	970 ± 188	921 ± 226	0.689	952 ± 194	941 ± 135	0.631	946 ± 282	1020 ± 160	0.588
**OTUs**	533 ± 106	542 ± 115	0.89	570 ± 132	534 ± 85	0.593	550 ± 217	572 ± 87	0.262
**Shannon**	4.98 ± 0.85	5.13 ± 0.75	0.759	5.45 ± 1.39	5.3 ± 0.56	0.808	4.91 ± 0.96	5.68 ± 0.72	0.151
**Simpson**	0.9 ± 0.05	0.91 ± 0.05	0.631	0.91 ± 0.11	0.93 ± 0.02	0.522	0.89 ± 0.05	0.94 ± 0.04	0.064

**Table 2:** Significance tests of the effects of adding the bacterial consortium on the overall bacterial community structure at three time points using three statistical approaches

	MRPP		ANOSIM		Adonis	
	δ	***P***	R	***P***	F	***P***
**Placebo-7 vs BMC-7**	0.413	0.151	0.079	0.211	1.225	0.152
**Placebo-14 vs BMC-14**	0.407	0.080	0.198	0.072	1.388	0.093
**Placebo-21 vs BMC-21**	0.367	**0.018**	0.305	**0.036**	1.592	**0.023**

Alignment of the sequences of the four inoculated strains with those of the high-throughput sequencing database revealed that close sequences of four strains were not detected at the end of experiment.

### Comparison of 16S rRNA gene composition among samples from different treatments

**Fig. 3:**
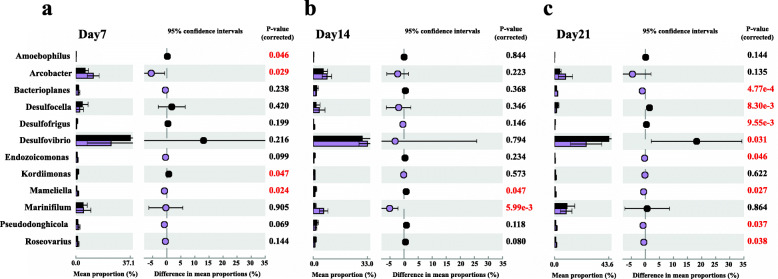
Subsystems enriched or depleted with the bacterial genera between the placebo and BMC groups. Data from days 7 (**a**), 14 (**b**) and 21 (**c**). Subsystems overrepresented in the added bacterial consortium treatment community with positive (negative) differences between proportions are indicated by different colors (black is placebo group; purple is BMC group). Red font: *P* < 0.05

To determine which microbial populations in the coral samples were affected by the bacterial consortium, the significantly changed genera were identified from their relative abundances using an extended error bar in Statistical Analysis of Metagenomics Profiles (STAMP; Fig. 3). The bacterial consortium significantly altered the relative abundance of several genera in the microbial community of the coral sample. On day 7, four genera were significantly altered, more than half of which were Proteobacteria (Fig. 3a). Relative abundances of the genera *Arcobacter* (11.75%) from the class Epsilonproteobacteria and *Mameliella* (1.45%) from Alphaproteobacteria were significantly increased (*P* < 0.05), while *Amoebophilus from* Bacteroidetes and *Kordiimonas* from Alphaproteobacteria were significantly decreased (*P* < 0.05; Fig. 3). On day 14, the relative abundance of the genus *Marinifilum* (6.3%) in the class Bacteroidetes significantly increased (*P* < 0.01), and that of the genus *Mameliella* significantly decreased (*P* = 0.047; Fig. 3b). On day 21, the relative abundances of eight genera from the phylum Proteobacteria changed significantly (Fig. 3). These included *Pseudodonghicola*, *Mameliella*, and *Roseovarius* from Alphaproteobacteria and *Bacterioplanes* and *Endozoicomonas* from the order Oceanospirillales of Gammaproteobacteria, which all showed significant increases in the relative abundances (*P* < 0.05; Fig. 3), while those of genera *Desulfocella*, *Desulfofrigus* and *Desulfovibrio* from Deltaproteobacteria were significantly decreased (*P* < 0.01).

### Relationship between coral bacterial community and physiological parameters

**Fig. 4:**
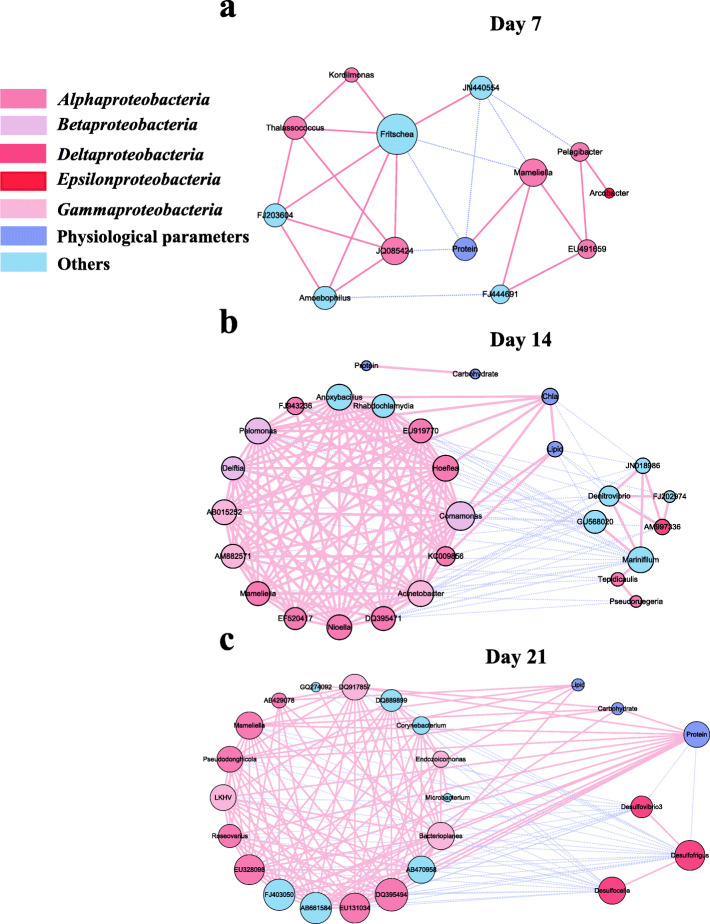
Co-occurrence patterns of markedly changing genera and physiological parameters of the coral. Network analysis was conducted on data from days 7 (**a**), 14 (**b**), and 21 (**c**). Each connection indicates a strong significant correlation, with Spearman’s correlation coefficient ≥ 0.5 and *P <* 0.05. Each node represents a microbial genus, and the size is proportional to the node connectivity. Each edge represents a linkage between two co-occurring nodes, and the color represents a correlation (red is positive; purple is negative). All nodes are labeled with annotated genera, which are colored at the phylum level except Proteobacteria, which is at the class level)

Co-occurrence network analysis was used to explore potential interactions between the markedly altered genera and physiological parameters of the coral. Genera with r ≥ 0.5 to other genera or physiological parameters were analyzed (Fig. 4). The bacterial consortium both directly or indirectly affected the physiological state of coral.

Network analysis of data from day 7 samples showed that there was a simple interaction between bacterial genera and coral physiology, with 25 significant correlations (*P* < 0.05) present, 18 of which were positive correlations (Table 3). Four genera were directly related to the physiological parameters of the corals. Protein concentration was negatively correlated with three genera and positively correlated with one genus (*Mameliella* from Alphaproteobacteria; Fig. 4a). Of the three negatively correlated genera, *Fritschea* from Chlamydiae was the key hub with the highest connectivity (degree = 8), displaying positive correlation with *Kordiimonas* and *Thalassococcus* from Alphaproteobacteria, and *Amoebophilus* from Bacteroidetes. However, *Mameliella*, the only genus positively correlated with protein concentration, was indirectly positively correlated with *Arcobacter* from Epsilonproteobacteria through EU491659 and *Pelagibacter* from Alphaproteobacteria (Fig. 4a). Moreover, the relative abundances of *Mameliella* and *Arcobacter* increased significantly (Fig. 3), and their average proportions were the highest, indicating that these two genera may affect protein content of the coral.

**Table 3:** General topological properties of the network analysis using the differential genera between the Placebo and BMC groups on days 7, 14 and 21

Network metrics	Day 7	Day 14	Day 21
**Number of Nodes**	13	28	24
**Total number of edges**	25	171	156
**Number of positive correlations**	18	121	114
**Number of negative correlations**	7	50	42

Network analysis of the data from day 14 samples revealed that only a few genera were positively related to the physiological parameters of the coral, although the network was complex. Specifically, the network analysis yielded 171 significant correlations (*P* < 0.05), 121 of which were positive (Table 3). Among them, 15 were directly correlated with the physiological parameters of the coral, with nine being positive correlations. Chlorophyll-a and lipids were significantly positively correlated with the genus *Comamonas* and negatively correlated with the genus *Denitrovibrio* (Fig. 4b). Moreover, *Comamonas* and *Denitrovibrio* were significantly positively correlated with the genera *Mameliella* and *Marinifilum*, respectively, suggesting that *Mameliella* and *Marinifilum* may have played key roles in the changes of chlorophyll-a and lipid contents of the coral.

Network analysis of the data from day 21 samples showed a complex interaction between the markedly altered genera and coral physiology, most of which were positive effects. The analysis yielded 156 significant correlations (*P* < 0.05), with 114 being positive correlations (Table 3). Among them, 24 were directly correlated with the physiological parameters of the corals, with 22 being positive correlations. Protein concentration of the coral was negatively correlated with the genera *Desulfovibrio* and *Desulfofrigus*, and positively correlated with *Mameliella*, *Pseudodonghicola*, *Roseovarius* and *Bacterioplanes*. Carbohydrate and lipids contents were significantly positively correlated with *Mameliella* and *Pseudodonghicola*, indicating that these two genera may contribute to the total energy reserves of corals (Fig. 4c). Additionally, the genus *Endozoicomonas* (class Gammaproteobacteria, family Oceanospirillaceae), which is regarded as a beneficial bacterium of coral, did not directly interact with the protein, lipid, or carbohydrate contents of the coral, but could influence these physiological parameters by building close correlations with other genera. For example, *Endozoicomonas* was significantly positively correlated with the genus *Bacterioplanes* and negatively correlated with *Desulfofrigus* (Fig. 4c). Thus, *Endozoicomonas* is crucial to the total energy reserves of coral.

## Discussion

Inoculating coral with microbes has yielded promising outcomes to support the feasibility of enhancing the resilience and/or resistance of coral by exogenously adding bacterial consortia [35–37, 41]. The present study was the first to investigate microbial community compositions and coral physiology after periodically adding a BMC consortium to coral. The study demonstrated that addition of an exogenous BMC consortium can change the coral-associated microbial communities and elevate coral energy reserves, and this may aid growth of the coral. Consistent with the results of Rosado et al., the results of the present study further support that the coral microbiome can be changed, and reasonable manipulation can improve the health of the coral [35]. Although the molecular mechanisms associated with such improvements remain unexplored.

Proteobacteria is the dominant phylum of coral-associated bacteria that are commonly found in *Pocillopora damicornis* [42], and this finding extends to most coral microbial communities, including reef-building and soft coral [43–45]. After periodically adding a BMC consortium to the coral, the relative abundance of Deltaproteobacteria in the BMC group significantly decreased compared with that of the placebo group at the end of the experiment. Moreover, the bacterial community structure was apparently changed on day 21, which is consistent with the findings of Santos et al. [41]. Changes in the bacterial community structure of coral may be due to a dynamic relationship existing between symbiotic microorganisms and environmental conditions, which results in selection of the most advantageous coral holobiont [30]. Additionally, manipulation of the coral microbial community by addition of an exogenous bacterial consortium may influence the coral community structure by increasing or decreasing the abundances of beneficial or harmful microorganisms.

Four experimental strains belonged to Alphaproteobacteria and Gammaproteobacteria, but the relative abundances of two classes did not change significantly during the experiment. Furthermore, no sequences from the inoculated strains were found in the BMC group at the end of the experiment, which indicated that the four inoculated strains might not be established in the coral microbiome. These results are consistent with previously reported findings that exogenously inoculated microbes were not detected in experimental coral following inoculation with a bacteria consortium or Symbiodiniaceae cocktail [35, 46]. Moreover, other research related to aquaculture activities showed that specific probiotics can improve growth performance, promote nutrient utilization and inhibit adherence of pathogenic bacteria, but cannot be successfully colonized [47, 48]. Unsuccessful colonization of the bacterial consortium in term of coral growth in the current research might be associated with the short-term addition period or concentration of the inoculated strains [49]. In addition, the added bacterial consortium probably did not play a direct role in the coral-associated bacterial community through establishing themselves in the coral holobiont, but may have impacted the coral microbial community by indirectly affecting other closely related microbial members in the coral.

Relative abundances of *Arcobacter* and *Mameliella* increased markedly in the BMC group compared with those of the placebo group on day 7, and these two genera were the first and second most abundant genera among all significantly altered genera on day 7 (Fig. 3). The genus *Mameliella* belongs to the family Rhodobacteraceae of Alphaproteobacteria. *Mameliella alba* is a probiotic bacterium involved in nutrient metabolism, aromatic compound degradation, and production of prebiotics, and may promote growth of Symbiodiniaceae through nitrogen fixation and vitamin production [50–54]. In the current study, *Mameliella* was significantly positively correlated with protein, lipid and carbohydrate contents of the coral samples, indicating that this genus may be closely related to the added bacterial consortium and might benefit the total energy reserve of the coral, most likely through nutrient uptake and utilization [51]. High abundances of *Arcobacter* (Epsilonproteobacteria) have been found in various diseased marine organisms, including necrotic sponges [55], moribund oysters [56], and coral with white plague disease and black band disease [57–59], as well as in healthy coral samples [60–62]. *Arcobacter* contributed indirectly to coral protein content reserves in the present study. Therefore, some *Arcobacter* may also be abundant in healthy coral hosts and might benefit coral growth and development.

The genus *Marinifilum* is involved in sulfur coupling and the carbon cycle in environments with variable redox conditions and oxygen availability [63], and is likely related to the primary hydrolysis and fermentation of spirulina by catalyzing the initial hydrolysis and fermentation of proteins, carbohydrates and lipids to diverse volatile fatty acids [64]. The relative abundance of *Marinifilum* increased significantly in the coral samples treated with BMC consortium, and was indirectly negatively correlated with coral lipid content on day 14. The decreased lipid content in BMC-treated coral compared with that of the placebo groups may have been because *Marinifilum* participated in lipid catabolism. The sulfate-reducing bacteria *Desulfocella*, *Desulfofrigus* and *Desulfovibrio* decreased significantly on day 21 [65–67]. *Desulfovibrio* spp. have been consistently documented in black band disease, which actively kills coral tissue, leaving the exposed skeleton behind [68]. *Desulfovibrio* is considered a secondary and necessary pathogen required for initiation and development of black band disease in coral hosts, and causes degradation of the underlying coral tissues, mainly by producing sulfide within the band as a byproduct of dissimilatory sulfate reduction [67–71]. Thus, the significant decrease in *Desulfovibrio* at the end of the experiment suggested that addition of the bacterial consortium could improve coral health.

The proportion of potential probiotics in the BMC group, including *Mameliella*, *Bacterioplanes* and *Endozoicomonas*, increased significantly on day 21. *Endozoicomonas*, a beneficial symbiont, contributes to cycling carbohydrates and providing proteins to the coral host [72]. Furthermore, this bacterial genus can protect its host from the coral pathogen *Vibrio coralliilyticus* by metabolizing dimethylsulfoniopropionate into dimethyl sulfide [73]. The relative abundance of *Endozoicomonas* increased significantly and was indirectly positively correlated with the total energy index of the coral, including the protein, lipid, and carbohydrate contents. Thus, adding a bacterial consortium may improve the resistance and competitiveness of a coral by shifting the structure of the coral-associated microbial community to increase the total energy reserves for coral growth and/or protect the host from pathogens.

Corals have two nutrient sources: symbiotic algal photosynthesis and heterotrophic polyp feeding [8]. However, the chlorophyll-a content and Fv/Fm values (which were determined from Symbiodiniaceae photosystem functions via pulse-amplitude-modulated fluorometry measurements) showed no significant changes between the BMC and placebo groups. NOV-1 and NOV-C, two strains of the bacteral consortium used to inoculate the coral, belonged to the genus *Salipiger* (family Rhodobacteraceae, class Alphaproteobacteria). Although this genus is only reported to produce exopolysaccharide and degrade the phthalate ester, some genera of Rhodobacteraceae can fix nitrogen [74–77]. The other two strains of the bacterial consortium, P1 and SP4, belonged to the genera *Salinicola* and *Phytobacter* respectively, and some species of these genera were reported to be resistant to heavy metals and promote plant growth [78, 79]. Based on growth characteristics in the current study, *Salinicola* and *Phytobacter* may play their beneficial role by solubilizing phosphate. This preliminary finding suggests that addition of the bacterial consortium may enhance coral nutrients availability in a heterotrophic way rather than via *Symbiodiniaceae* photosynthesis in the absence of environmental pressure and providing that respiration activity remains the same.

In summary, the application of beneficial microorganisms in coral is promising, and additional mid- and long-term realistic laboratory and well-controlled field pilot experiments are essential to unveil the modulated symbiotic mechanisms, microbiome dynamics, connectivity with other organisms and ecological improvements and outcomes, to indicate and define risk assessment boundaries and provide a safe framework to be applied in light of specific coral reef conditions and urgency for protection and rehabilitaion [80, 81].

## Materials and methods

### Coral collection and rearing

Five live adult *Pocillopora damicornis* colonies were collected from the Luhuitou fringing reefs, Sanya Bay (18° 12′ N, 109° 28′ E), on May 8, 2017 and were deployed in water tanks at the laboratory of Tropical Marine Biological Research Station in Hainan, China. All colonies were subsequently fragmented into smaller coral nubbins (~ 5 cm high and weighing 12 g) and were fixed onto a circular ceramic base with aquarium glue. All nubbins were reared in 1000-L tanks containing running seawater at the local sea temperature for 1 week to recover and stabilize.

### Preparation of pure bacterial cultures

The bacterial consortium comprised four pure bacterial genera: *Yangia* (NOV-1), *Roseobacter* (NOV-C), *Phytobacter* (SP4) and *Salinicola* (P1), which were previously isolated from different coral tissues (Table S1) and frozen at − 80 °C with 25% glycerin. NOV-1 and NOV-C, both containing the *nif*H gene, were initially isolated and purified on Ashby medium (10 g mannitol, 0.2 g KH_2_PO_4_, 0.2 g MgSO_4_‧7H_2_O, 0.2 g NaCl, 0.1 g CaSO_4_, 5 g CaCO_3_, and 18 g of agar in 1 L distilled water at pH 7.0; Additional file 2). P1 and SP4 were initially isolated and purified on phytase-screening medium (15 g D-glucose, 5 g NH_4_NO_3_, 2 g CaCl_2_, 0.5 g KCl, 0.5 g MgSO_4_‧7H_2_O, 0.01 g FeSO_4_‧7H_2_O, 0.01 g MnSO_4_‧7H_2_O, 25 g NaCl, 4 g Na-phytate (sterilized separately), and 18 g of agar in 1 L distilled water, 7.0 pH) [82]. NOV-1 and NOV-C were recovered with nutrient agar medium (4 g tryptone, 0.2 g yeast extract, 0.48 g Tris, 0.01 g K_2_HPO_4_, 0.001 g FeSO_4_‧7H_2_0, 4.8 g MgSO_4_‧7H_2_O, 4 g MgCl‧6H_2_O, 0.56 g KCl, 25 g NaCl, 2 mL glycerinum, and 18 g of agar in 1 L distilled water, at pH 8.2) [83]. P1 and SP4 were recovered with phytase-screening medium. All four strains were fermented with Luria-Bertani (LB) medium (10 g tryptone, 5 g yeast extract, 10 g NaCl, and 18 g of agar in 1 L seawater, at pH 7.0).

The four pure strains were individually inoculated into 250 mL LB medium and incubated at 28 °C for 24 h. Cell numbers of each strain were estimated using a hemocytometer and light microscope. When all strains reached 10^8^ cells mL^− 1^, the cultures were centrifuged at 8000 rpm for 10 min. The cell pellets were washed three times in 0.22-μm-filtered seawater (FSW) and resuspended to 1 × 10^9^ cells mL^− 1^ in 25 mL of FSW. All four stains were evenly mixed to make the bacterial consortium (BMC). The final concentration of the added bacterial consortium contained 10^6^ cells mL^− 1^ of each isolate. In the placebo group, BMC was replaced with FSW.

### Experimental design and sampling

**Fig. 5:**
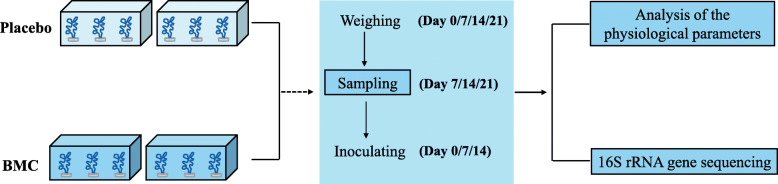
Experimental design flowchart. Placebo and BMC groups, which each comprised two aquariums containing 12 coral nubbins, were used to explore the effects of addition of a bacterial consortium on coral physiology and microbial community structure. Buoyancy weighing and coral sampling were conducted every 6 days, and three corals were collected per aquarium at time point. After each sampling, the BMC group was inoculated with the bacterial consortium, and the placebo group received FSW. Water-flow exchange with the outside of the aquarium was stopped for 24 h after each inoculation

The experimental system included four independent 14-L aquariums (Fig. 5). Each aquarium contained 12 L 0.5-μm FSW and had an individual water pump (AtmanAT-302 with 450 L h^− 1^ water flow; Zhongshan, China) to form a 24-h circulating loop. Each aquarium could exchange seawater with a sump containing 0.5-μm FSW. The seawater flow rate was 100 mL min^− 1^ for each aquarium, providing a half-fold volume replacement every hour. The aquariums received 250 μmol photons m^− 2^ s^− 1^, with a 12-h light/12-h dark photoperiod, and the seawater temperatures were held at 28 °C. Following stabilization of the experimental system, the 48 coral nubbins were randomly distributed among the four aquariums and allowed to acclimatize for 1 week. Five nubbins per aquarium were then marked for measuring the coral skeletal growth through weighing for buoyancy. The four aquariums were randomly divided into two groups (placebo and BMC), with two aquariums per group due to limitations in experimental conditions. The well-established bacterial consortium was used to inoculate the two BMC aquariums on day 0, 7 and 14, respectively. And the three sampling points during experiment are on day 7, day 14 and day 21, respectively. After each inoculation, the water-flow exchange with the outside of all aquariums (placebo and BMC aquariums) was stopped for 24 h to maintain a setting bacterial concentration and allow the bacterial consortium enough time to act; the individual water pump inside each aquarium are still running to form a 24-h circulating loop. Marked nubbins were weighed for buoyancy every 6 days, then three unmarked samples were collected from each aquarium, and the next inoculation was added. Each sampled nubbin was wrapped in sterile zinc sheet and hammered into homogenates, which were mixed well, divided into two parts and preserved at − 80 °C. One part was used for measurement of the physiological parameters of the coral and the other part was used for high-throughput sequencing. The samples for molecular analysis were rapidly added to DNA protector (Takara, Japan) before storage. On the penultimate day of the experiment, the maximum quantum yield of the PSII photochemistry (Fv/Fm) was measured at 6:00 am, 12:00 noon and 18:00 pm, reflecting the potential photosynthetic efficiency of Symbiodiniaceae.

### Determination of physiological parameters of the coral

Tissue biomass was measured by drying 1 g of sample to constant dry weight (24 h, 60 °C) and burning it in a muffle furnace at 450 °C for 6 h. The difference between dry and burned weight was the ash-free dry weight, which was standardized to the wet weight of the sample [84].

For determination of chlorophyll-a concentration, 0.5 g sample was lysed in 1 ml of 4 °C 90% acetone and shaken three times for 10 s at 1-min intervals on ice. Samples were extracted for 2 h in the dark at 4 °C, then centrifuged at 10,000 rpm for 2 min to remove cellular debris. The absorbance of the supernatant was measured via enzyme-labeled instrument (ELIASA; wavelengths: 630 nm, 647 nm and 664 nm) to quantify the chlorophyll-a concentration as per Jeffery et al. [85].

Total lipids were extracted five times from 1 g sample in a 2:1 chloroform/ methanol solution, sonicated briefly (200 w, 1 min), then vortexed. Samples were centrifuged at 10,000 rpm for 1 min and the supernatants were transferred into a 10-ml centrifuge tubes. All supernatants were washed in 2 mL 0.88% KCl and centrifuged at 4000 rpm for 5 min to remove the supernatant, then washed three times in a 1:1 methanol/water solution. The extract was dried to a constant weight at 37 °C under a stream of pure nitrogen gas and standardized to the ash-free dry weight [84, 86]. Total protein and carbohydrates were extracted from the same sample and measured by colorimetric determination at 562 nm and 485 nm, respectively. Briefly, 10 μL phenylmethanesulfonyl fluoride (PMSF, 100 mM) and 1 mL radio-immunoprecipitation assay (RIPA) buffer were added to 1 g sample. The resulting slurries were vortexed five times for 5 s each, then centrifuged at 10,000 rpm for 2 min. The supernatants were transferred to a fresh 2-mL tube, then preserved at − 80 °C for later protein and carbohydrate quantification. One subsample of the supernatants was used for protein quantification using the bicinchoninic acid (BCA) method with bovine serum albumin as a standard [87] and the determination conducted as per the protocol of the BCA Protein Assay Kit (Beyotime, Shanghai, China). A second subsample was used for carbohydrate quantification using the phenol-sulfuric acid method with D-glucose as a standard [88]. Briefly, the supernatants were diluted 10 times with 0.9% NaCl and vortexed thoroughly. The diluents (200 μL) were transferred into a new 10-mL tube, and 10 μL of 80% phenol followed by 1 mL sulfuric acid were quickly added. The tube was shaken well in a shaker for 10 min and incubated in an oven at 40 °C for 30 min. The reaction mixtures were cooled to room temperature and measured using ELISA at 485 nm. Protein and carbohydrate concentrations were also standardized to the ash-free dry weight. Gross energy was calculated using the combustion enthalpies for protein (− 23.9 KJ g^− 1^), carbohydrate (− 17.5 KJ g^− 1^) and lipid (− 39.5 KJ g^− 1^) [89].

Calcification rates were determined using the buoyant weight technique [90] and were standardized to ash-free dry weight. The maximum quantum yields of the PSII photochemistry (Fv/Fm) were measured on the living corals using diving PAM fluorometry [41], and each coral colony was randomly measure five times and averaged, with nine replicates per group.

### DNA extraction and amplification

Samples were removed from − 80 °C storage, thawed at room temperature, then centrifuged at 5000 rpm for 1 min to remove the DNA protectant. DNA was extracted from the samples using an E.Z.N.A. Soil DNA Kit (Omega, USA) according to the manufacturer’s instructions. DNA concentration and purity were assessed using a NanoDrop 1000 (Thermo Fisher Scientific, USA). Bacterial 16S rRNA genes were amplified by PCR from genomic template DNA in 25-μL reactions (with three technical replicates and a negative control) containing 12.5 μL Taq PCR mix enzyme (Takara, Japan), 1 μL DNA template, and 0.5 μL each primer (10 μM). The primer were 341F (5′-CCTACGGGNGGCWGCAG-3′) and 805R (5′-GACTACHVGGGTATCTAATCC-3′) [91], which were attached to 6mer barcodes in advance and supplemented with 10.5 μL of ddH_2_O. PCR conditions comprised an initial denaturation step at 95 °C for 5 min, 35 cycles of 95 °C for 30 s, 56 °C for 30 s, and 72 °C for 32 s, followed by a final elongation step at 72 °C for 5 min. The resulting amplification products were checked on a 1.2% agarose gel to ensure they were 470 bp. From the DNA concentration and molecular weight, PCR products from all samples were pooled in equal proportions, then purified using a gel extraction kit (Omega) per the manufacturer’s protocol. Purified products were sent to GENEWIZ Co. (Suzhou, China) for library preparation and sequencing on the Illumina HiSeq system.

### Bioinformatics and statistical analyses

Partial sequences of the 16S rRNA gene were processed using the QIIME pipeline, version 2010 [92]. Forward and reverse sequences were merged and split into samples based on the barcode sequences. Sequences were then denoised, chimeras were removed, and OTUs were defined at 97% similarity. OTUs were annotated using the EzBiocloud reference database [40]. Singletons were removed from the OTU tables.

Differences in physiological parameters of the coral were analyzed via one-way analysis of variance in SPSS 21.0 (IBM, USA) and visualized using Origin 8.1 software (OriginLab, USA). SPSS 21.0 was also used to analyze the differences in α-diversity indexes (i.e., Chao 1, OTU, Shannon and Simpson) between the placebo and BMC groups. Independent-sample t-tests were used to determine whether the data were normally distributed, and the Mann-Whitney U test was used for data that were not normally distributed. Beta-diversity was analyzed with the Data Analysis Pipelines of the Institute for Environmental Genomics (http://www.ou.edu/ieg/tools/data-analysis-pipeline). The coral microbial community composition and PCoA plot was visualized using R ggplot2 and the reshape2 package (version 3.6.2). Bacterial community differences at the genus level were determined using Welch’s t-test in the STAMP (Statistical Analysis of Metagenomic Profiles) software package (version 2.1.3) [93]. Differential genera were visualized based on effect size > 0.2 and *P* < 0.05. Sequence alignments were performed using rBLAST package (version R 4.0.3).

Co-occurrence network analysis was performed using R 3.6.2 and Cytoscape 3.3.0 to analyze correlations between the physiological parameters and all differential genera between the placebo and BMC groups at different time points (Additional file 4) [94]. The resulting network topology was determined by calculating the pairwise Spearman’s correlation coefficients. Nodes represent genera or physiological parameters; edges represent the correlations between them (red is positive correlation; purple is negative correlation). Node size was proportional to the number of edges linked to it.

## Conclusions

This study investigated how addition of a well-established bacterial consortium influenced coral-associated microbial communities and the physiological status of coral. Although bacterial community α-diversity did not significantly change with the addition of the bacterial consortium, the BMC group exhibited distinct bacterial community structures compared with those of the placebo group at the end of the experiment. Moreover, coral in the BMC group had more energy reserves and higher calcification rates compared with the placebo group. The bacterial consortium contributed to the coral health, possibly through shifting the coral-associated bacterial community structure, as reflected in the increasing the proportion of potentially beneficial bacteria in BMC samples. In addition, the unchanged photosynthesis-related parameters (Fv/Fm and chlorophyll-a) implied that the bacterial consortium contributed to the stored energy and calcification rate of the coral by enhancing the nutrient availability from the heterotrophic pathway rather than via Symbiodiniaceae photosynthesis, or by triggering beneficial microbial rearrangements (as observed in our data) in the absence of environmental pressure. However, the mechanism of action and long-term functional stability of the inoculated bacterial consortium require further study.

## Supplementary Information

**Additional file 1: Figure S1.** Average protein concentrations (a), carbohydrate concentrations (b) and lipid concentrations (c) in the placebo (white bars) and BMC (gray bars) group on days 7, 14, and 21. All averages are standardized to grams of ash-free dry tissue weight (gdw), *n*=6. * and ** indicate significant differences at *P*<0.05 and *P*<0.01, respectively. Bars represent the standard deviation of the mean. **Figure S2.** (a) Chlorophyll-a concentration of BMC (triangle) versus placebo (circle) and are standardized to the ash-free dry weight of the sample. (b) The maximum quantum yields of the PSII photochemistry (Fv/Fm) was determined at the day before the end of experiment. Number of coral fragments: Fv/Fm, n=4 and chlorophyll-a, n=6. **Figure S3.** Principal coordinates analysis (PCoA) of *Pocillopora damicornis* microbiome in the placebo (red) and BMC (blue) group on days 7 (b), 14 (c), 21 (d) and three time points (a), based on Bray-Curtis dissimilarity index, *n* = 6. **Table S1.** Characteristics of the bacteria strains used to generate the inoculation bacterial consortium. nt: nucleotides.

**Additional file 2 **The *nif*H gene sequences of the two strains.

**Additional file 3.** Changes in the relative abundance at class level at different sampling times.

**Additional file 4.** The differential genera between the placebo and BMC groups at different sampling times.

## Data Availability

The sequence data generated and analyzed in this study are available at NCBI (https://www.ncbi.nlm.nih.gov) under accession numbers (PRJNA669106).
