# Correction to: Shifting the microbiome of a coral holobiont and improving host physiology by inoculation with a potentially beneficial bacterial consortium

**DOI:** 10.1186/s12866-021-02219-w

**Published:** 2021-05-19

**Authors:** Ying Zhang, Qingsong Yang, Juan Ling, Lijuan Long, Hui Huang, Jianping Yin, Meilin Wu, Xiaoyu Tang, Xiancheng Lin, Yanying Zhang, Junde Dong

**Affiliations:** 1 CAS Key Laboratory of Tropical Marine Bio-resources and Ecology, Guangdong Provincial Key Laboratory of Applied Marine Biology, South China Sea Institute of Oceanology, Chinese Academy of Sciences, Guangzhou, 510301 China; 2 Southern Marine Science and Engineering Guangdong Laboratory (Guangzhou), Guangzhou, 511458 China; 3 Tropical Marine Biological Research Station in Hainan, Chinese Academy of Sciences and Hainan Key Laboratory of Tropical Marine Biotechnology, Sanya, 572000 China; 4 Innovation Academy of South China Sea Ecology and Environmental Engineering, Chinese Academy of Sciences, Guangzhou, 510301 China; 5 University of Chinese Academy of Sciences, Beijing, 100049 China; 6 Ocean School, Yantai University, Yantai, 264005 China

**Correction to: BMC Microbiol 21, 130 (2021)**

**https://doi.org/10.1186/s12866-021-02167-5**

Following the publication of the original article [1], we have been notified of a mistake in the order of affiliations.

The original article has been corrected.
