# Supplementary material for: Shifting the microbiome of a coral holobiont and improving host physiology by inoculation with a potentially beneficial bacterial consortium
Source: BMC Microbiol. 2021 Apr 28;21:130. doi: 10.1186/s12866-021-02167-5 (PMC8082877; doi:10.1186/s12866-021-02167-5)
Supplement: Supplementary file 1 — Additional file 1: Figure S1. Average protein concentrations (a), carbohydrate concentrations (b) and lipid concentrations (c) in the placebo (white bars) and BMC (gray bars) group on days 7, 14, and 21. All averages are standardized to grams of ash-free dry tissue weight (gdw), n=6. * and ** indicate significant differences at P<0.05 and P<0.01, respectively. Bars represent the standard deviation of the mean. Figure S2. (a) Chlorophyll-a concentration of BMC (triangle) versus placebo (circle) and are standardized to the ash-free dry weight of the sample. (b) The maximum quantum yields of the PSII photochemistry (Fv/Fm) was determined at the day before the end of experiment. Number of coral fragments: Fv/Fm, n=4 and chlorophyll-a, n=6. Figure S3. Principal coordinates analysis (PCoA) of Pocillopora damicornis microbiome in the placebo (red) and BMC (blue) group on days 7 (b), 14 (c), 21 (d) and three time points (a), based on Bray-Curtis dissimilarity index, n = 6. Table S1. Characteristics of the bacteria strains used to generate the inoculation bacterial consortium. nt: nucleotides. [file 12866_2021_2167_MOESM1_ESM.docx]

Shifting the microbiome of a coral holobiont and improving host physiology by inoculation with a potentially beneficial bacterial consortium

Ying Zhang^1,2,4,5,6^, Qingsong Yang^1,2,5,6^, Juan Ling^1,2,5^, Lijuan Long^1,2,4,5^, Hui Huang^1,2,4,5^, Jianping Yin^1,2^, Meilin Wu^1,2^, Xiaoyu Tang^1,6^, Xiancheng Lin^1,6^, Yanying Zhang^3*^, Junde Dong^1,2,4,5*^

^1^CAS Key Laboratory of Tropical Marine Bio-resources and Ecology, Guangdong Provincial Key Laboratory of Applied Marine Biology, South China Sea Institute of Oceanology, Chinese Academy of Sciences, Guangzhou 510301, China.

^2^Southern Marine Science and Engineering Guangdong Laboratory (Guangzhou), Guangzhou 511458, China.

^3^Ocean School, Yantai University, Yantai 264005, China

^4^Tropical Marine Biological Research Station in Hainan, Chinese Academy of Sciences and Hainan Key Laboratory of Tropical Marine Biotechnology, Sanya 572000, China.

^5^Innovation Academy of South China Sea Ecology and Environmental Engineering, Chinese Academy of Sciences, Guangzhou 510301, China.

^6^University of Chinese Academy of Sciences, Beijing 100049, China.

*Corresponding author

E–mail: [dongjd@scsio.ac.cn](mailto:dongjd@scsio.ac.cn); zhyanying@163.com

Tel/Fax: +86-20-89107830

Table S1 Characteristics of the bacterial strains used to generate the inoculation bacterial consortium. nt: nucleotides.

| Bacterial identity | GenBank accession | Isolation source | Geographical description | Coordinates | Closest relative | % identity from BLAST of X nt of the 16S rRNA gene |
| --- | --- | --- | --- | --- | --- | --- |
| NOV-1 | MW159356 | *Pocillopora woodjonesi* | Gaven Reef | 10°12′4″N,  114°13′25″E | *Salipiger* sp. | 99.63% from 1360 nt |
| NOV-C | MW159357 | *Ctenactis crassa* | Gaven Reef | 10°12′4″N,  114°13′25″E | *Salipiger* sp. | 99.70% from 1354 nt |
| P1 | MW159359 | *Porites lutea* | Fiery Cross Reef | 9°33′00″N,  112°53′24″E | *Salinicola* sp. | 99.51% from 1436 nt |
| SP4 | MW159358 | *Goniastrea edwardsi* | Johnson South Reef | 9°44′6″N,  114°16′56″E | *Phytobacter* sp. | 99.79% from 1440 nt |


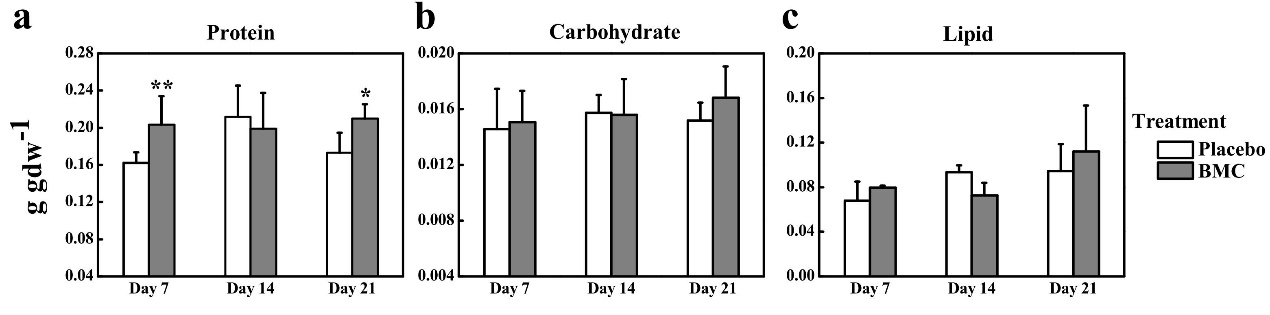


Figure S1. Average protein concentrations (a), carbohydrate concentrations (b) and lipid concentrations (c) in the placebo (white bars) and BMC (gray bars) group on days 7, 14, and 21. All averages are standardized to grams of ash-free dry tissue weight (gdw), *n*=6. * and ** indicate significant differences at *P*＜0.05 and *P*＜0.01, respectively. Bars represent the standard deviation of the mean**.**


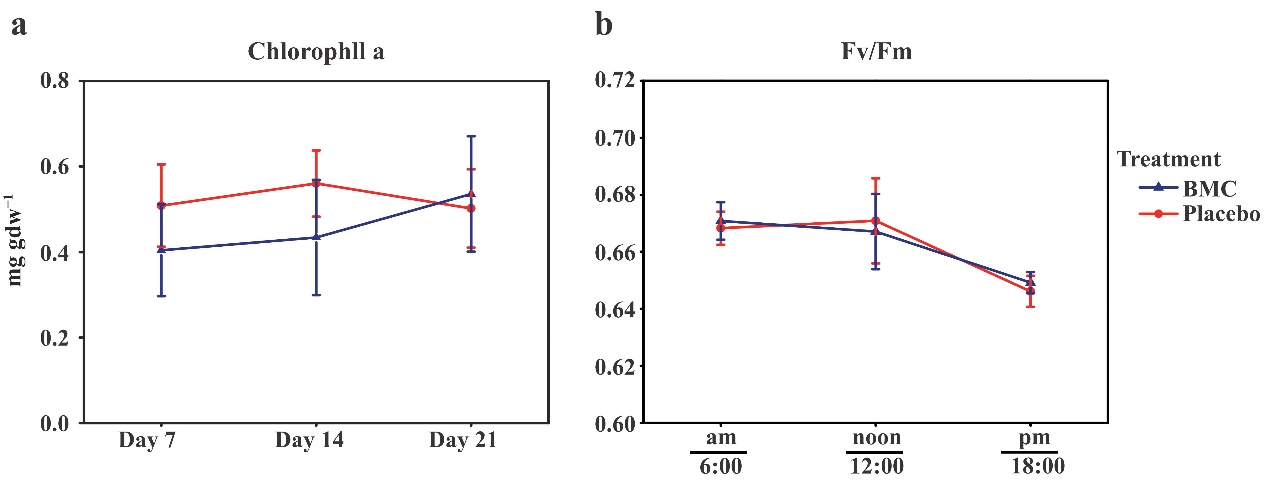


Figure S2. (a) Chlorophyll-a concentration of BMC ( blue triangle) versus placebo (red circle) and are standardized to the ash-free dry weight of the sample. (b) The maximum quantum yields of the PSII photochemistry (Fv/Fm) was determined at the day before the end of experiment. Number of coral fragments: Fv/Fm, *n*=4 and chlorophyll-a, *n*=6**.**

**
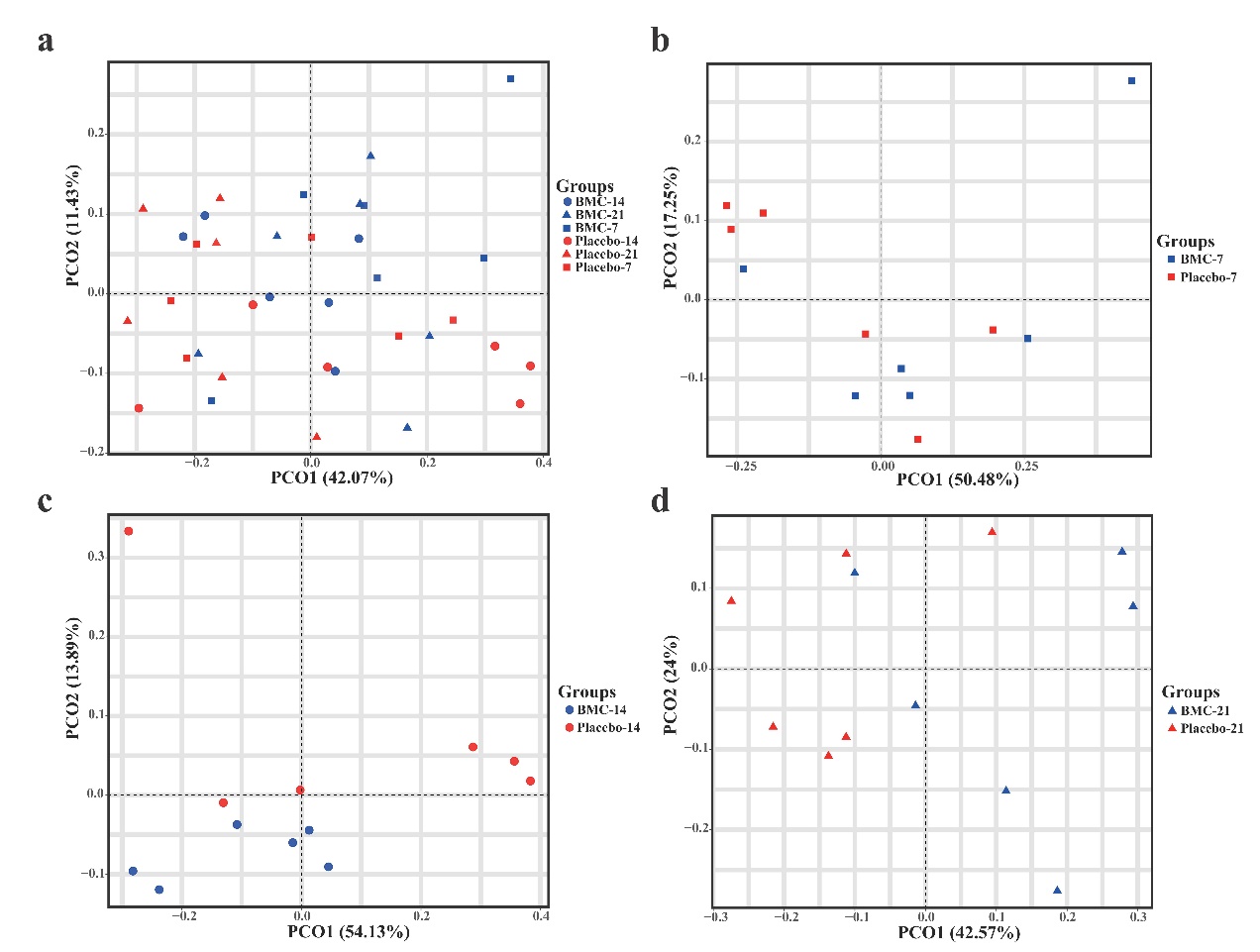
**

Figure S3. Principal coordinates analysis (PCoA) of Pocillopora damicornis microbiome in the placebo (red) and BMC (blue) group on days 7 (b), 14 (c), 21 (d) and three time points (a), based on Bray-Curtis dissimilarity index, *n*=6**.**
